# The main protease of SARS-CoV-2 downregulates innate immunity via a translational repression

**DOI:** 10.1038/s41392-023-01418-3

**Published:** 2023-04-13

**Authors:** Weifeng Liang, Ming Gu, Lingxiang Zhu, Ziqi Yan, Dominik Schenten, Shelby Herrick, Hongmin Li, Subodh Kumar Samrat, Jiapeng Zhu, Yin Chen

**Affiliations:** 1 School of Medicine & Holistic Integrative Medicine, Nanjing University of Chinese Medicine, Nanjing, Jiangsu 210023 China; 2 Department of Pharmacology and Toxicology, School of Pharmacy, The University of Arizona, Tucson, AZ 85721 USA; 3 Department of Immunology, School of Medicine, The University of Arizona, Tucson, AZ 85724 USA; 4 Jiangsu Key Laboratory for Pharmacology and Safety Evaluation of Chinese Materia Medica, School of Pharmacy, Nanjing University of Chinese Medicine, Nanjing, Jiangsu, 210023 China; 5 Jiangsu Joint International Research Laboratory of Chinese Medicine and Regenerative Medicine, Nanjing University of Chinese Medicine, Nanjing, Jiangsu, 210023 China

**Keywords:** Translational immunology, Innate immunity

**Dear Editor**,

SARS-CoV-2 is the etiological cause of COVID-19. RIG-I-like receptor (RLR) signaling pathway appeared to be responsible for SARS-CoV-2 induced IFN,^1^ a major antiviral pathway. IFN deficiency^2,3^ was identified to be a significant risk factor of severe COVID-19, highlighting an important role of IFN in the defense against SARS-CoV-2 and in the pathogenesis of severe COVID-19. Since SARS-CoV-2 is highly susceptible to IFN treatment,^3,4^ it has to evade host IFN-mediated immune surveillance to establish an active infection,

To identify viral regulators of IFN, we designed a screening strategy, in which MAVS, the common adaptor protein, was overexpressed to initiate the downstream IFN signaling. Each viral protein encoded by SARS-CoV-2 was introduced into the system to examine its effect on MAVS-IFN signaling. We found that NSP5, the main protease of SARS-CoV-2, is among the five viral proteins (NSP1, 3, 5, ORF6, and 8) that significantly repressed MAVS-IFN (Supplementary Fig. 1a). In contrast, NSP5 (C145A), an enzymatic inactive NSP5, could not inhibit MAVS-induced ISRE reporter activity (Supplementary Fig. 1b). Furthermore, NSP5 was able to inhibit IFN promoter reporter (PRDIII) and this inhibition was mostly restored by a protease inhibitor of NSP5 (GC376) (Supplementary Fig. 1c). Next, we extended the study of reporter assay (Supplementary Fig. 1) to cellular RLR signaling pathway. MAVS overexpression induced MAVS aggregation (a pre-requisite for MAVS activation) and STAT1 phosphorylation (Fig. 1c), confirming that MAVS overexpression indeed activated the downstream IFN pathway. We further tested if overexpression of upstream RNA sensors (MDA5 and RIG-I) or downstream signaling proteins (TBK1 and IRF3) could also activate IFN pathway. Indeed, an overexpression of MDA5 (Fig. 1a), RIG-I (Fig. 1b), TBK1 (Fig. 1d) and IRF3 (Fig. 1e) robustly induced STAT1 phosphorylation. At the mRNA level, we tested MAVS (Fig. 1f) or TBK1 (Fig. 1g) overexpression and found that both could increase the expression of selected interferon stimulated genes (or ISGs) (e.g. IFNβ, and ISG15). The cellular protease activity of transfected NSP5 was verified by flipGFP model system (Supplementary Fig. 2), in which an inactive flipGFP could be activated by a specific cleavage of NSP5 recognition site. Indeed, transfected NSP5, but not NSP5 (C145A), elicited a strong protease activity as demonstrated by a robust GFP signal (Supplementary Fig. 2). Consistent with the results from the reporter gene assay (Supplementary Fig. 1), co-transfection with NSP5, but not with NSP5 (C145A), significantly repressed STAT1 activation (Fig. 1a-e, Supplementary Fig. 3) and MAVS aggregation (Fig. 1c). Additionally, increased expressions of ISGs (IFNβ and ISG15) were almost completely repressed by NSP5, but not by NSP5 (C145A) (Fig. 1f, g). We also tested two other common IFN stimuli: poly (I:C) and Sendai virus (SeV) infection. Indeed, their downstream IFN signaling pathways were also repressed by NSP5, but not by NSP5 (C145A) (Supplementary Fig. 4a, b). Taken together, NSP5 significantly repressed RLR pathway in a protease-dependent manner.

**Fig. 1:**
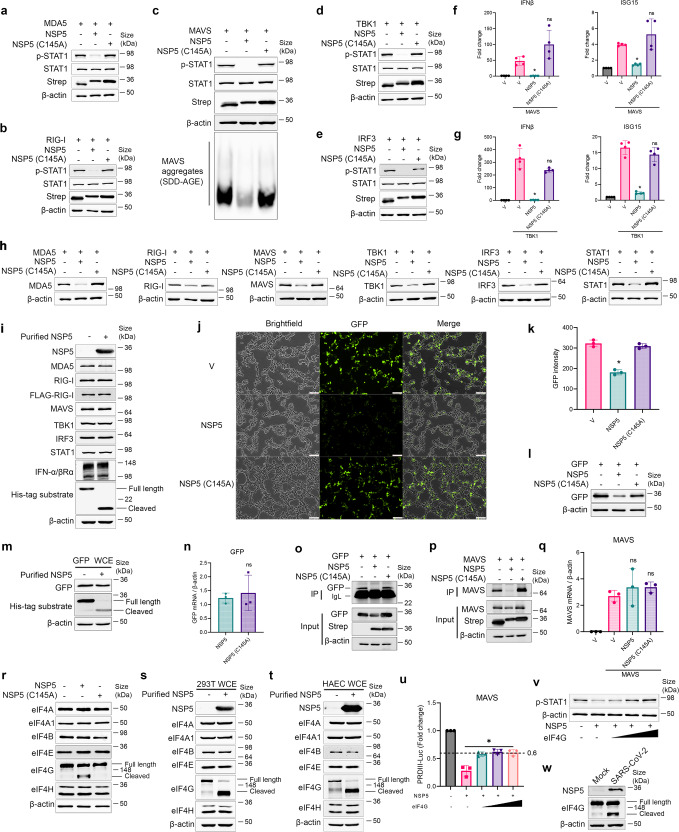
NSP5 repressed RLR-IFN pathway by affecting the translation. Immunoblotting analysis of p-STAT1, STAT1 when cells were transfected with MDA5 (**a**), RIG-I (**b**), MAVS (**c**), TBK1(**d**), and IRF3 (**e**) along with either NSP5 or NSP5 (C145A). Oligomerized MAVS (aggregates) was analyzed by SDD-AGE. Strep-tag (in the control vector, NSP5 or NSP5 (C145A)) was measured to ensure their proper expressions. β-actin was used as a loading control. Cells were transfected with MAVS (**f**) or TBK1 (**g**) along with NSP5 or NSP5 (C145A). Total RNA was extracted and analyzed by RT-qPCR with primers specific for IFNβ and ISG15. *: *P* < 0.05. ns no significance. *n* = 4. **h** Immunoblotting analysis of ectopically expressed protein level of MDA5, RIG-I, MAVS, TBK1, IRF3 and STAT1 in 293 T cells along with NSP5 or NSP5 (C145A). **i** 293 T whole cell extracts (WCEs) were incubated with purified NSP5 at RT for 1 h. Since endogenous MDA5, RIG-I and IFN receptor were low, WCE from FLAG-MDA5, FLAG-RIG-I and IFNαR1 + R2 transfected cells were used. MDA5, RIG-I, MAVS, TBK1, IRF3, STAT1 and IFN-α/βRα were analyzed by immunoblotting. His-tag linked control peptides were spiked in as an internal control. **j** Fluorescence images of 293 T cells transfected with GFP along with NSP5 or NSP5 (C145A). Scale bar = 100 μm. **k** A quantification of GFP fluorescence intensity (Excitation 485 nm, Emission 535 nm). **l** Immunoblotting analysis of GFP expression in the cells transfected with GFP along with NSP5 or NSP5 (C145A). **m** WCE of cells transfected with GFP was incubated with purified NSP5. His-tag linked control peptides were spiked in as an internal control. **n** qPCR measurement of GFP RNA in the cells co-transfected with NSP5 or NSP5 (C145A). SUnSET analysis of newly synthesized GFP (**o**) and MAVS (**p**). IgL Light chain of IgG. **q** qPCR measurement of MAVS RNA in the cells co-transfected with vector only (V), NSP5 or NSP5 (C145A). **r** A screen identified endogenous translation initiation factors that were targeted by an overexpressed NSP5 in 293 T cells. Immunoblotting analysis of translation initiation factors in 293 T cells (**s**) or in HACEs’ whole cell extracts (**t**) cleaved by purified NSP5. **u** Cells were transfected with MAVS, NSP5, PRDIII-Luc, Renilla-Luc and an increasing amount of eIF4G. A dual luciferase assay was performed. **v** Cells were transfected with MAVS, NSP5, and an increasing amount of eIF4G. Total cellular protein was collected and p-STAT1 was analyzed by immunoblotting analysis. **w** Immunoblotting analysis of NSP5, eIF4G in HAECs infected with SARS-CoV-2 (MOI = 1)

Serendipitously, we noticed a significant reduction of protein level of each co-transfected immune component in the presence of NSP5, but not NSP5 (C145A) (Fig. 1h). We first tested the hypothesis that NSP5 accelerates proteins degradation via either proteasomal or lysosomal pathway. Cells were treated with proteasome inhibitor ((R)- or (S)-MG132) or lysosome inhibitor (Bafilomycin A1 or BFA). Neither MG132 nor BFA could restore NSP5-mediated protein reduction of MAVS, MDA5, RIG-I, TBK1, or STAT1 (Supplementary Fig. 5a–e). Thus, neither proteasomal nor lysosomal pathway was responsible for NSP5-mediated protein reduction. Since NSP5 is a protease, we then tested if NSP5 could directly cleave (or degrade) any of these proteins by incubating cell lysates with purified NSP5 protein. As a positive control, a peptide (His-tag linked substrate) containing NSP5 recognition site was spiked in these lysates. Although purified NSP5 successfully cleaved the positive control peptide to produce a lower-MW band, it did not cleave (or degrade) any protein in RLR pathway (Fig. 1i) or affected MAVS aggregates in three different doses (Supplementary Fig. 5f). There was another report suggesting that NSP5 might cleave N-terminal 10 amino acid (AA) from RIG-I.^5^ Since our transfected RIG-I has N-terminal FLAG-tag, this 10-AA cleavage, if exist, would remove the FLAG-tag, thereby reducing the signal of FLAG-RIG-I or making it completely disappear. However, using either RIG-I or FLAG antibody, we did not observe such cleavage (Fig. 1i). Furthermore, in contrast to their finding that NSP5 specifically promoted K48 ubiquitination of MAVS,^5^ we found that NSP5, but not NSP5(C145A), reduced total ubiquitinated protein expression regardless of their links to wild-type, K48- or K63 ubiquitin (Supplementary Fig. 5g). Collectively, although co-transfected NSP5 reduced the protein expression of components in RLR pathway, this reduction was not caused by a direct cleavage or degradation mediated by NSP5 protein.

To our surprise, when co-transfected with NSP5, but not NSP5 (C145A), the brightness of GFP was significantly reduced (Fig. 1j, k). The repression appeared to be caused by a reduced level of GFP protein expression (Fig. 1l), but not by a direct GFP cleavage (Fig. 1m) or decreased GFP transcription (Fig. 1n). A SUnSET assay (Supplementary Materials and Methods), in which puromycin was used as a structural analog of aminoacyl tRNAs to label nascent polypeptide chains, was performed to verify the effect of NSP5 on protein translation. Indeed, puromycin-labeled newly synthesized GFP was completely inhibited by NSP5, but not by enzymatic deficient NSP5 (C145A) (Fig. 1o), confirming that NSP5-mediated reduction of GFP expression was controlled at the level of translation. The same observation was also made in the experiment of NSP5-mediated reduction of MAVS protein (Fig. 1p). Consistently, MAVS mRNA level was not affected by NSP5 (Fig. 1q). However, in contrast to NSP1 (a viral protein known to repress global protein translation via ribosomal inhibition), NSP5 appeared not to affect global protein translation in a SUnSET assay (Supplementary Fig. 5h). Altogether, these data strongly support a model in which NSP5 repressed selected protein translation.

To further elucidate potential mechanisms of translational regulation by NSP5, we tested if transfected NSP5 could cleave any component of eukaryotic translation initiation factor (eIF) complex (i.e. 4 A, 4A1, 4B, 4E, 4 G and 4H). Among all these factors, only eIF4G was cleaved (Fig. 1r). The cleavage site (LQ658.659GI) was further confirmed by a site mutation assay (Supplementary Fig. 6a). Additionally, purified NSP5 specifically cleaved eIF4G not only in 293T cells (Fig. 1s), but also in physiologically relevant human primary cells (Fig. 1t). We also found that NSP5 was co-localized with an Endoplasmic Reticulum marker, but not with a Golgi marker (Supplementary Fig. 6b), further supporting the function of NSP5 in translational regulation. To establish a cause and effect, we tested if eIF4G overexpression could restore NSP5-mediated repression of RLR-IFN. In this experiment, an ectopic expression of eIF4G restored NSP5-mediated ISRE reporter repression by up to approximately 60% (Fig. 1u). However, this restoration appeared not to be dose dependent. Furthermore, eIF4G overexpression restored NSP5-mediated p-STAT1 reduction by almost 100% (Fig. 1v). To further test if eIF4G can be cleaved in a setting of real SARS-CoV-2 infection, we infected human primary airway epithelial cells with live SARS-CoV-2. NSP5 was found to be highly expressed in the infected cells together with a cleaved eIF4G (Fig. 1w). Therefore, NSP5 appeared to specifically cleave eIF4G, which was likely responsible for its repressive function on the protein translation and RLR-mediated innate immunity. The identification of eIF4G as a cellular target of NSP5 may provide a possible explanation for its lack of significant impact on global protein translation (Supplementary Fig. 5h), as most of those newly synthetic proteins were likely made from translational elongation. Instead, NSP5 may preferentially affect those newly initiated proteins (e.g. IFN, some cytokines etc.) involving in immune responses to viral infection, which places NSP5 in a unique position to regulate viral evasion of host immunity in a more subtle manner than a full-blown translational repressor such as NSP1. Another intriguing question is if NSP5 affects viral translational initiation. Although this notion requires further investigation, mechanisms must be already in place for viruses to escape from NSP5’s inhibition as previously documented for NSP1 (Supplementary Fig. 5h). Nonetheless, NSP5 is likely a multi-functional protein and acts in concert with other viral proteins to dysregulate/repress host immunity in order to support viral life cycle.

In summary, we have discovered a novel translational mechanism that is likely responsible for NSP5-mediated IFN repression. eIF4G, a key component of translational initiation complex, was identified to be a specific NSP5 substrate in vitro as well as in a cell model of SARS-CoV-2 infection. Ectopic eIF4G expression was able to partly rescue NSP5-mediated repression of RLR-IFN pathway. Our finding provides a unique angle to the research of innate immunity against SARS-CoV-2 infection. Since IFN repression is a major mechanism of viral evasion and a determinant of severe COVID-19, our finding may reveal a new target (eIF4G) for research on COVID-19 pathogenesis.

## Supplementary information

Supplementary information

## Data Availability

The data that support the findings of this study are available from the corresponding author upon reasonable request.
