# Supplementary material for: The main protease of SARS-CoV-2 downregulates innate immunity via a translational repression
Source: Signal Transduct Target Ther. 2023 Apr 13;8:162. doi: 10.1038/s41392-023-01418-3 (PMC10098221; doi:10.1038/s41392-023-01418-3)
Supplement: Supplementary file 1 — Supplementary information [file 41392_2023_1418_MOESM1_ESM.docx]

Supplementary Materials for

The main protease of SARS-CoV-2 downregulates innate immunity via a translational repression

Weifeng Liang^1, 2^, Ming Gu^1, 2^, Lingxiang Zhu^2^, Ziqi Yan^2^, Dominik Schenten^3^, Shelby Herrick^2^, Hongmin Li^2^, Subodh Kumar Samrat^2^, Jiapeng Zhu^1^* and Yin Chen^2^*

^1^School of Medicine & Holistic Integrative Medicine, Nanjing University of Chinese Medicine, 210023, Nanjing, Jiangsu, China.

^2^Department of Pharmacology and Toxicology, School of Pharmacy, The University of Arizona, 85721, Tucson, AZ.

^3^Department of Immunology, School of Medicine, The University of Arizona, 85724, Tucson, AZ.

* Corresponding to: [ychen@pharmacy.arizona.edu](mailto:ychen@pharmacy.arizona.edu) or [zhujiapeng@hotmail.com](mailto:zhujiapeng@hotmail.com).

**This PDF file includes:**

Materials and Methods

Supplementary Fig. S1 to S6

Table S1

**Materials and Methods**

1. Plasmids

The plasmids containing different codon optimized SARS-CoV-2 open reading frames (ORFs) in the vector pLVX-EF1alpha-IRES-Puro were gifts from Dr. Krogan (University of California, San Francisco)^1^. FLAG-MDA5 and FLAG-RIG-I were gifts from Dr. Gack (University of Chicago). FLAG-TBK1 was a gift from Dr. Fitzgerald (University of Massachusetts). MAVS, FLAG-IRF3, FLAG-STAT1, HA-eIF4G, IFNAR1, IFNAR2 and FlipGFP were obtained from Addgene (Watertown, MA). HA-eIF4G mutants (Q658N, Q1127N, Q658N-Q1127N) were created using the QuikChange II XL Site-Directed Mutagenesis Kit (Agilent, Santa Clara, CA) following the manufacturer's instructions. The cleavage sites were based on a published work^2^.

1. Cell culture and virus

293T cells were obtained from American Type Culture Collection (ATCC, Manassas, VA). HeLa cell was a gift from Dr. Zhang (University of Arizona). Primary human airway epithelial cells (HAECs) were isolated from normal human bronchial tissues, which were obtained from the National Disease Research Interchange (Philadelphia, PA) by an approved protocol. Protease-dissociated cells were plated and maintained in immersed culture conditions until they reached confluence. Cells were infected with SARS-CoV-2 (Isolate USA_WA1/2020, <https://www.utmb.edu/wrceva>) at a multiplicity of infection (MOI) =1. All SARS-CoV-2 related work was conducted in a biological safety cabinet in a biosafety level 3 laboratory at The University of Arizona based on an approved protocol.

1. Luciferase reporter assay

293T cells were transfected with an ISRE-Luc or IFN (PRDIII)-Luc reporter plasmid along with a plasmid expressing each of different SARS-CoV-2 ORFs and a plasmid encoding MAVS. Renilla-Luc was used as an internal control for transfection efficiency. At 24-hour post-transfection, luciferase activity was measured by a Dual Luciferase Reporter Assay System (Promega, Madison, WI). The firefly luciferase activities were normalized to Renilla luciferase activities.

1. RNA extraction, cDNA synthesis and Real-Time quantitative PCR (qPCR)

Total RNA was extracted from cells using Direct-zol RNA Miniprep Kit (Zymo Research, Irvine, CA). cDNA was prepared from 1μg of total RNA and was then further diluted to 100 ul with 10mM Tris for the following procedures. Two microliters of diluted cDNA were analyzed using SYBR Green PCR Master Mix by a Veriti® Thermal Cycler (Thermo Fisher, Grand Island, NY). Primers were used at 0.25 μM. The primer is designed by us using the Primer 3 software. The relative mRNA amount in each sample was calculated based on the △△Ct method using housekeeping gene β- Actin. Results were calculated as fold induction over control. Primers are listed in Table S1.

1. Antibodies and western blot

Anti-strep tag (SAB2702215), anti-FLAG (M2; F1804) and anti-Puromycin (MABE343) were purchased from Sigma. Anti-His tag (2365), anti-HA tag (3724), anti-pSTAT1 (9167), anti-STAT1 (9176), anti-TBK1 (3013), anti-RIG-I (4520), anti-GFP (2955), anti-NSP5 (51661), anti-IRF3 (4302), anti-LC3B (2775) and Translation Initiation Complex Antibody Sampler Kit (4763, included eIF4A, eIF4A1, eIF4B, eIF4E, eIF4G, eIF4H) were purchased from Cell Signaling Technology (Danvers, MA). Anti-Calnexin (PA5-34754) was from Invitrogen (Waltham, MA). Anti-GM130 (ab52649) was from Abcam (Cambridge, UK). Anti-MAVS (sc-166583), anti-Nrf2 (sc-13032) anti-IFN-α/βRα (sc-7391) and anti-β-actin (sc-47778) were from Santa Cruz Biotechnology (Dallas, TX). Total cellular proteins were collected based on the methods described previously^3^. Equal protein loading was confirmed using anti-β-actin. The experiment was repeated at least three times.

1. GFP imaging

293T cells cultured on 6-well plates were transfected with a plasmid expressing eGFP, along with plasmids encoding NSP5 or NSP5 (C145A) for 24h. In a FlipGFP assay^4^, cells were transfected with a FlipGFP plasmid containing a specific NSP5 recognition site. The images were acquired by Nikon microscope (Nikon Eclipse Ti Microscope, Tokyo, Japan) using a 10x objective. And the GFP signals intensity was measured on a TECAN infinite M200 plate reader using emission filters for GFP fluorescence.

1. Fluorescence microscopy

HeLa cells were cultured on 2-well chamber slide (Nunc Lab-Tek). After transfection with NSP5 plasmid for 24h, cells were fixed with 4% paraformaldehyde for 10 min, and then permeabilized in 0.2% Triton X-100/PBS for 10 min at room temperature. After washing with PBST for 3 times, cells were blocked in 3% BSA/PBST for 1 hour, and then incubated with primary antibodies diluted in 1% BSA/PBST overnight. The cells were washed and followed by a fluorescently labeled secondary antibody (Chicken anti-Mouse IgG (H+L) Cross-Adsorbed Secondary Antibody, Alexa Fluor™ 488; Chicken anti-Rabbit IgG (H+L) Cross-Adsorbed Secondary Antibody, Alexa Fluor™ 594, Invitrogen). Supper resolution images were examined using a microscope (Zeiss Elyra S.1) equipped with 63× oil objective. The images were processed using ZEN 3.6 (blue edition) software.

1. In vitro protease cleavage assay

Cells were harvested and lysed in buffer (20 mM Tris PH 7.4, 100 mM NaCl, 1 mM DTT, 1 mM EDTA). Whole cell extracts (WCEs) and purified recombinant NSP5 were incubated at room temperature (RT) for 1h. Reactions were stopped by the addition of 4× SDS sample buffer and subsequent boiling at 95°C for 5 min, and samples were analyzed by SDS-PAGE and immunoblotting analysis.

1. Cloning and purification of NSP5 and its positive control substrate

Codon-optimized gene sequence of the SARS-CoV-2 NSP5 was synthesized and replaced by the SARS-CoV main protease sequence in the Addgene plasmid 61692 through seamless cloning technology by GeneUniversal. The construct contained a modified pGEX-6P-1 backbone to generate authentic N-terminus of the NSP5 through autocleavage, and a C-terminal His-tag GPHHHHHH to facilitate purification. The His-tag could be cleaved by the HRV 3C protease to generate authentic NSP5 C-terminus. The expression plasmid was transformed into *E. coli* Rosetta (DE3) cells and then cultured in Sper broth medium containing 100 μg/ml ampicillin at 37 °C. When the cells were grown to an optical density at 600 nm of 0.6, 0.5 mM IPTG was added to the cell culture to induce the expression at 16 °C. After 16 h, the cells were collected by centrifugation at 7,000g. The cell pellets were resuspended in lysis buffer (20 mM Tris-HCl pH 8.0, 150 mM NaCl), lysed by sonication, and then centrifuged at 20,000 rpm for 30 min. The supernatant was loaded onto Ni-NTA affinity column (Qiagen) and washed in the resuspension buffer containing 20 mM imidazole. The His-tagged NSP5 was eluted by 300 mM of imidazole in lysis buffer. Human rhinovirus 3C protease was added to remove the C-terminal His tag. The NSP5 was further purified by size-exclusion chromatography using 75 Superdex column. Peak fractions were collected and pooled together. The purified NSP5 was stored in a buffer containing 20 mM Tris-HCl, pH 8, 150 mM NaCl, 1 mM DTT. For the NSP5 positive control substrate, codon-optimized nanoluciferase gene sequence encoding nanoluciferase with GGGGG[ERELNGGAPIKS]GGGG(KTSAVLQSGFRKME)GGGG RRRRSAGGGSGGG sequence inserted between nanoluciferase residues 51 and 52 was synthesized and inserted between the Nco1 and Xho1 sites of the pET28a vector. Purification of the substrate was carried out similarly as described above for the NSP5, with the following modifications. Upon elution from the Ni-NTA column, the protein was dialyzed in a buffer containing 20 mM Tris-HCl pH 8.0, 150 mM NaCl and 1mM DTT. Dialyzed proteins were stored in -80°C.

1. Semidenaturing detergent agarose gel electrophoresis (SDD-AGE)

SDD-AGE was performed according to a published protocol^5^. In brief, fresh whole cell extracts were completed with 4x loading dye consisting of 20% glycerol, 8% SDS, and bromophenol blue in 2x TAE buffer, and loaded onto a vertical 1.5% agarose gel. Migration was performed in the running buffer (1 × TAE and 0.1% SDS) for 35 minutes with constant voltage of 100V at 4 °C. After electrophoresis, the proteins were transferred to nitrocellulose membranes (Bio-Rad) for immunoblotting.

1. Surface sensing of translation (SUnSET)

The assay was modified from a published method^6^. Briefly, transfected cells were treated with puromycin (2.5ug/mL) for 6h. Exactly 300 µg of total cell lysates were brought to 150 µl with IP lysis buffer (Thermo Fisher, 87787) and incubated with 2.5 µl of anti-puromycin antibody (Millipore, MABE343) overnight at 4° C under end-over-end rotation. Approximately 70 µl Protein G Dynabeads (Thermo Fisher, 10004D) were then incubated with the sample-antibody complex for 2h at RT under rotation. Beads were washed three times with PBS containing 0.05% Tween-20. Samples were denaturing eluted with loading buffer and then subjected to immunoblot.

1. SARS-CoV-2 infection

Human ACE2 expression construct in adenovirus type 5 vector (Ad-hACE2-eGFP) (BEI resources, NR-52390) were used to infect HAECs first at MOI=4 to enhance viral replication in HAECs. Efficient gene expression of hACE2 was confirmed by eGFP fluorescent density using Nikon Ti fluorescence microscopy. 16 hours after Ad-hACE2 transduction, cells were infected with SARS-CoV-2 and mock at MOI=1 for 24 hours. Mock was deactivated SARS-CoV-2 that heated at 95 degrees for 5 min. Cells were collected for protein analysis as described.

1. Statistical analysis

Experimental groups were compared using a two-sided Student’s t test, with significance level set as P ＜ 0.05. When data were not distributed normally, significance was assessed with the Wilcoxon matched-pairs signed-ranks test, and P＜ 0.05 was considere­­­­d to be significant.

**References**

1 Gordon, D. E. *et al.* A SARS-CoV-2 protein interaction map reveals targets for drug repurposing. *Nature* **583**, 459-468 (2020).

2 Koudelka, T. *et al.* N-Terminomics for the Identification of In Vitro Substrates and Cleavage Site Specificity of the SARS-CoV-2 Main Protease. *Proteomics* **21**, e2000246 (2021).

3 Chen, Y. *et al.* Rhinovirus induces airway epithelial gene expression through double-stranded RNA and IFN-dependent pathways. *Am J Respir Cell Mol Biol* **34**, 192-203 (2006).

4 Zhang, Q. *et al.* Designing a Green Fluorogenic Protease Reporter by Flipping a Beta Strand of GFP for Imaging Apoptosis in Animals. *J Am Chem Soc* **141**, 4526-4530 (2019).

5 Halfmann, R. & Lindquist, S. Screening for amyloid aggregation by Semi-Denaturing Detergent-Agarose Gel Electrophoresis. *J Vis Exp.* (17) e838 (2008).

6 Schmidt, E. K., Clavarino, G., Ceppi, M. & Pierre, P. SUnSET, a nonradioactive method to monitor protein synthesis. *Nat Methods* **6**, 275-277 (2009).

7 Banerjee, A. K. *et al.* SARS-CoV-2 Disrupts Splicing, Translation, and Protein Trafficking to Suppress Host Defenses. *Cell* **183**, 1325-1339 e1321 (2020).

**
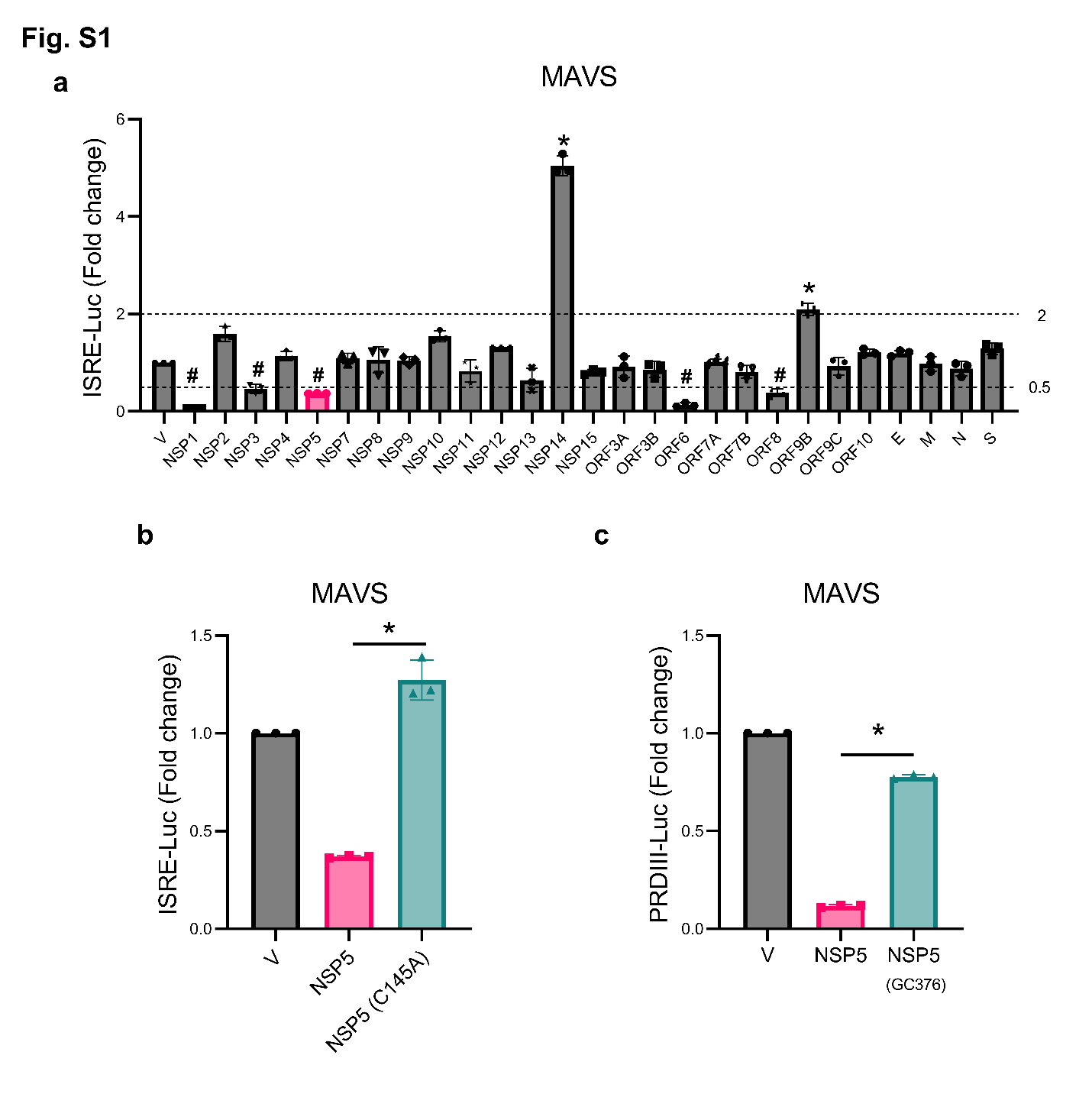
Supplementary Fig. S1: NSP5 repressed innate anti-SARS-CoV-2 signaling via a protease dependent mechanism.** *and #: P < 0.05. n=3. **a** 293T cells were transfected with MAVS, ISRE-luc, Renilla-luc along with each of different SARS-CoV-2 ORFs and vector control (V). A dual luciferase assay was performed. Fold induction was calculated as each ORF/ V. An increase by at least 2 folds or decrease by at least half was further used as a cut-off. **b** Cells were transfected with MAVS, ISRE-Luc, Renilla-Luc along with NSP5 or its catalytically dead mutant NSP5 (C145A). **c** Cells were transfected with MAVS, PRDIII-Luc and NSP5 in the presence or absence of GC376.


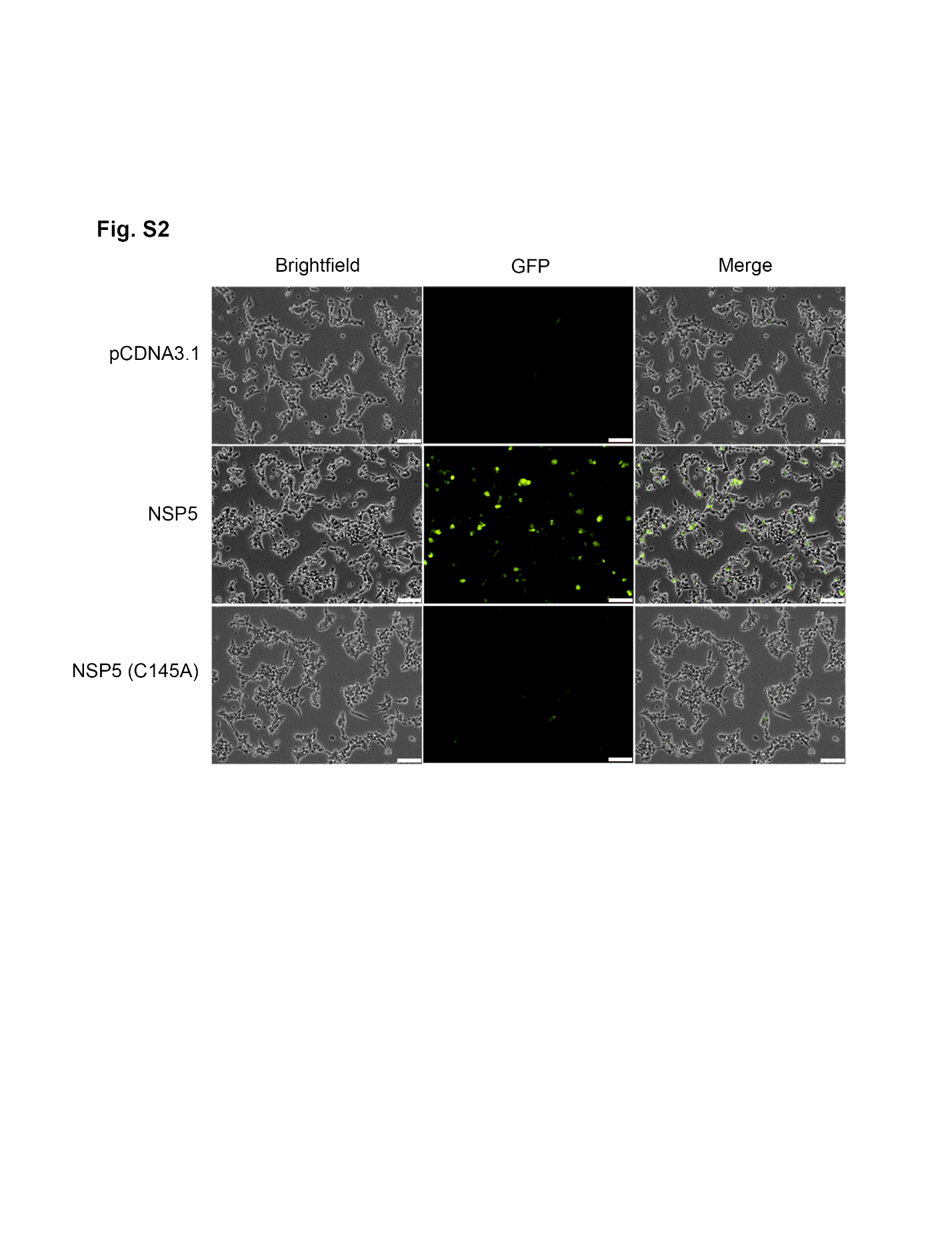
­

**Supplementary Fig. S2** FlipGFP-based NSP5 activity reporter was used to measure protease activity of transfected NSP5 in 293T cells. BF: bright field. Merge: a merged image of both bright field and fluorescent field. Scale bar = 100 μm.


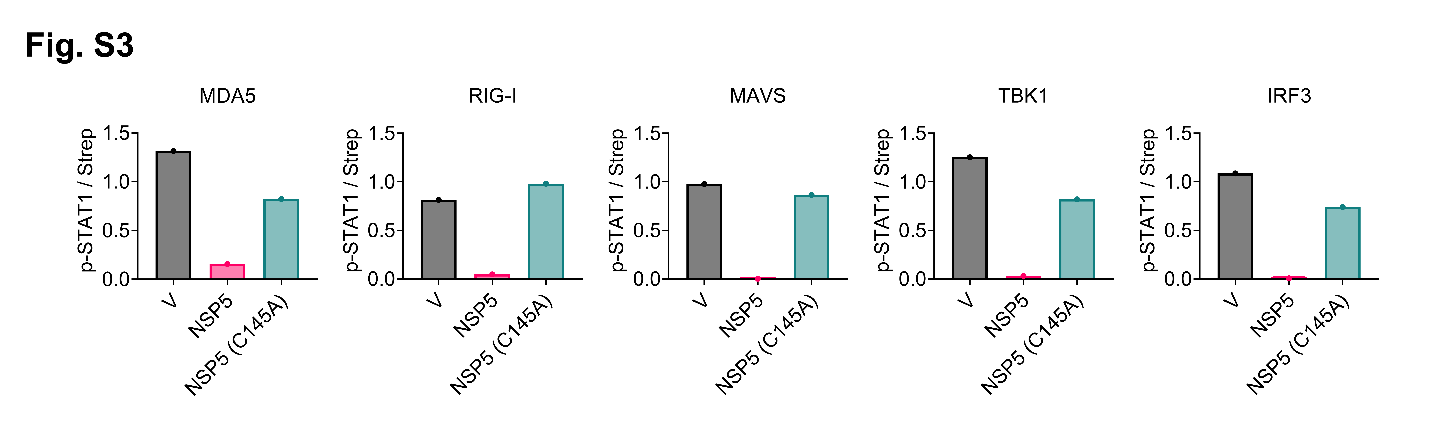


**Supplementary Fig. S3** Quantification of western blot analysis of p-STAT1 presenting in figure 1 a-e. Band intensity was measured with densitometric analysis and normalized to the intensity of Strep-tag (surrogate for the expression level of co-transfected vector only, NSP5 or NSP5 (C145A)).

**
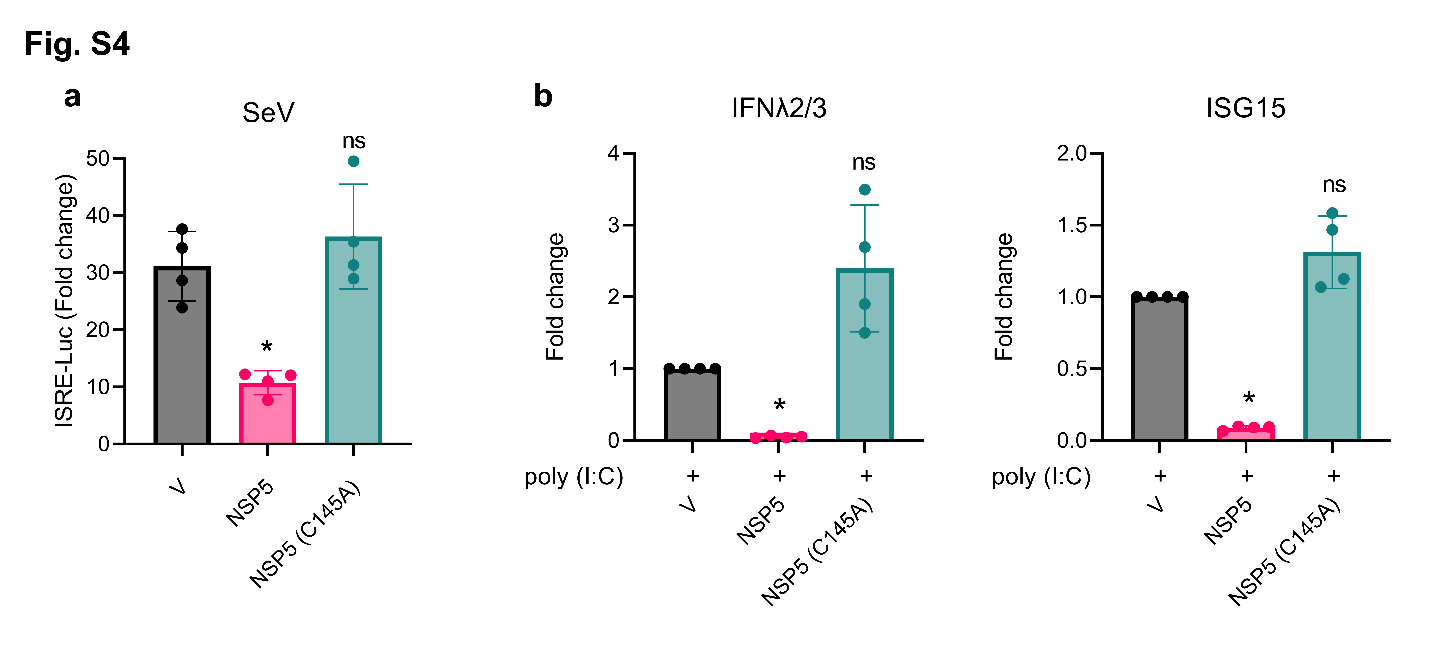
**

**Supplementary Fig. S4 a** 293T cells were transfected with ISRE-Luc, Renilla-Luc along with Vector only (V), NSP5 or its catalytically dead mutant NSP5 (C145A). Twenty‐four hours later, the cells were infected with Sendai Virus (SeV), and 24 h after stimulation, the cell lysate was collected for dual luciferase assay. **b** qPCR measurement of poly (I:C) induced IFN signals (IFNλ2/3 and ISG15) in the 293T cells transfected with V, NSP5 or NSP5 (C145A). RIG-I was ectopically expressed as the receptor of poly (I:C). *: P < 0.05. n=4. ns: not significant when compared with vector only.

**
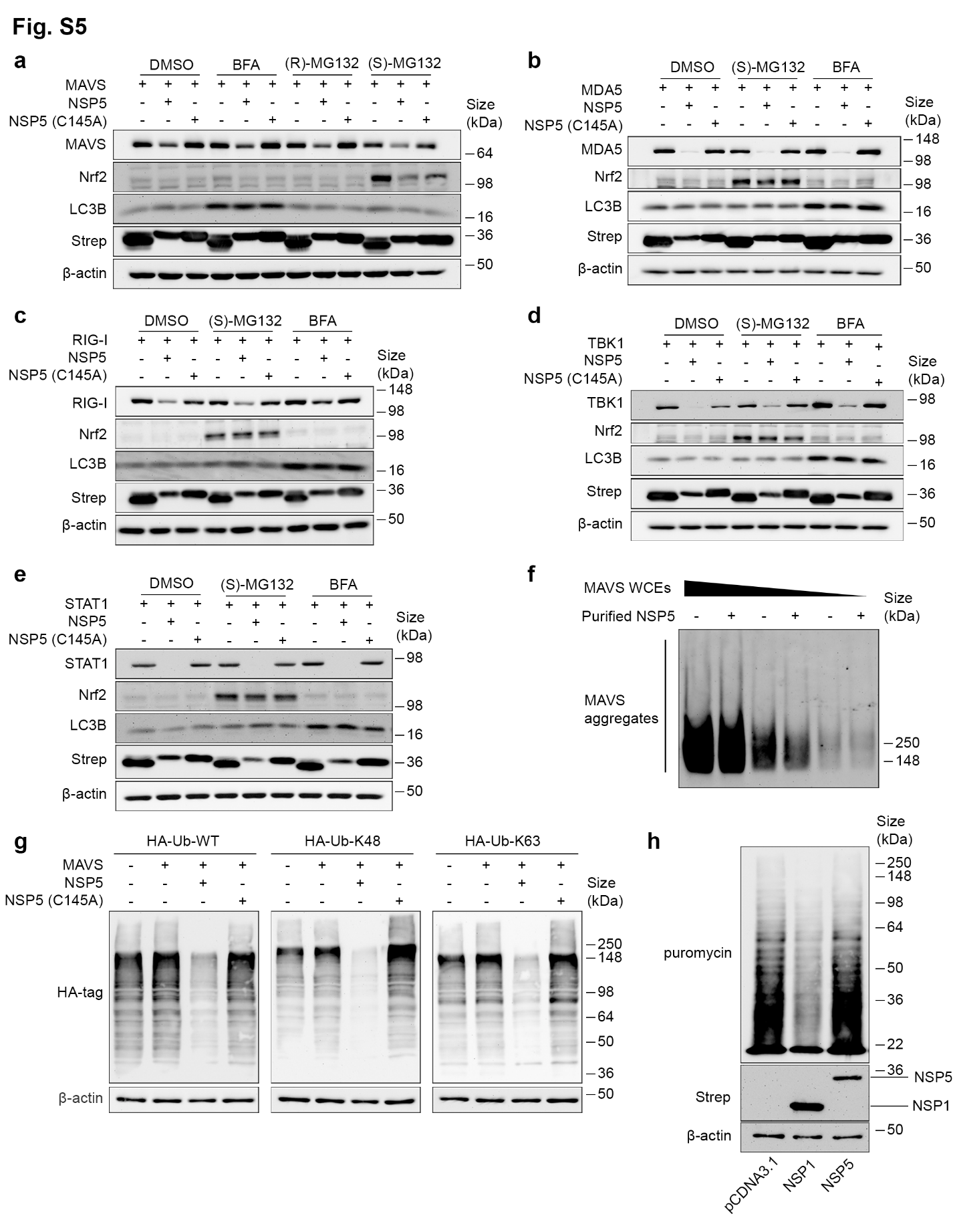
**

**Supplementary Fig. S5 a-e** Immunoblotting analysis of ectopically expressed MAVS (**a**), MDA5 (**b**), RIG-I (**c**), TBK1 (**d**), and STAT1 (**e**) in 293T cells along with NSP5 or NSP5 (C145A) and treated with dimethylsulfoxide (DMSO), MG132 (10 μM), or Bafilomycin A1 (BFA, 1 μM) for 10 hours before harvested. LC3B and Nrf2 served as MG132 or BFA treatment marker, β-actin was used as a loading control. **f** 293T cells were transfected with MAVS. Decreasing amounts of WCEs (60ug, 20ug, 7ug) were incubated with 12ug of purified NSP5 at RT for 1h. WCEs were resolved by SDD-AGE to analyze MAVS aggregates. **g** Immunoblotting analysis of ectopically expressed indicated HA tagged ubiquitin plasmids and MAVS with the expression of NSP5 or the NSP5 (C145A). HA-Ub-WT: wildtype ubiquitin. HA-Ub-K48: ubiquitin with K48 mutation. HA-Ub-K63: ubiquitin with K63 mutation. **h** The 293T cells were transfected for 20 h and puromycin labeled for 1 hour. Puromycin incorporation was determined by immunoblotting using anti-puromycin antibody. Strep-tagged NSP1^7^ and NSP5 proteins were detected by anti-Strep antibody.

**
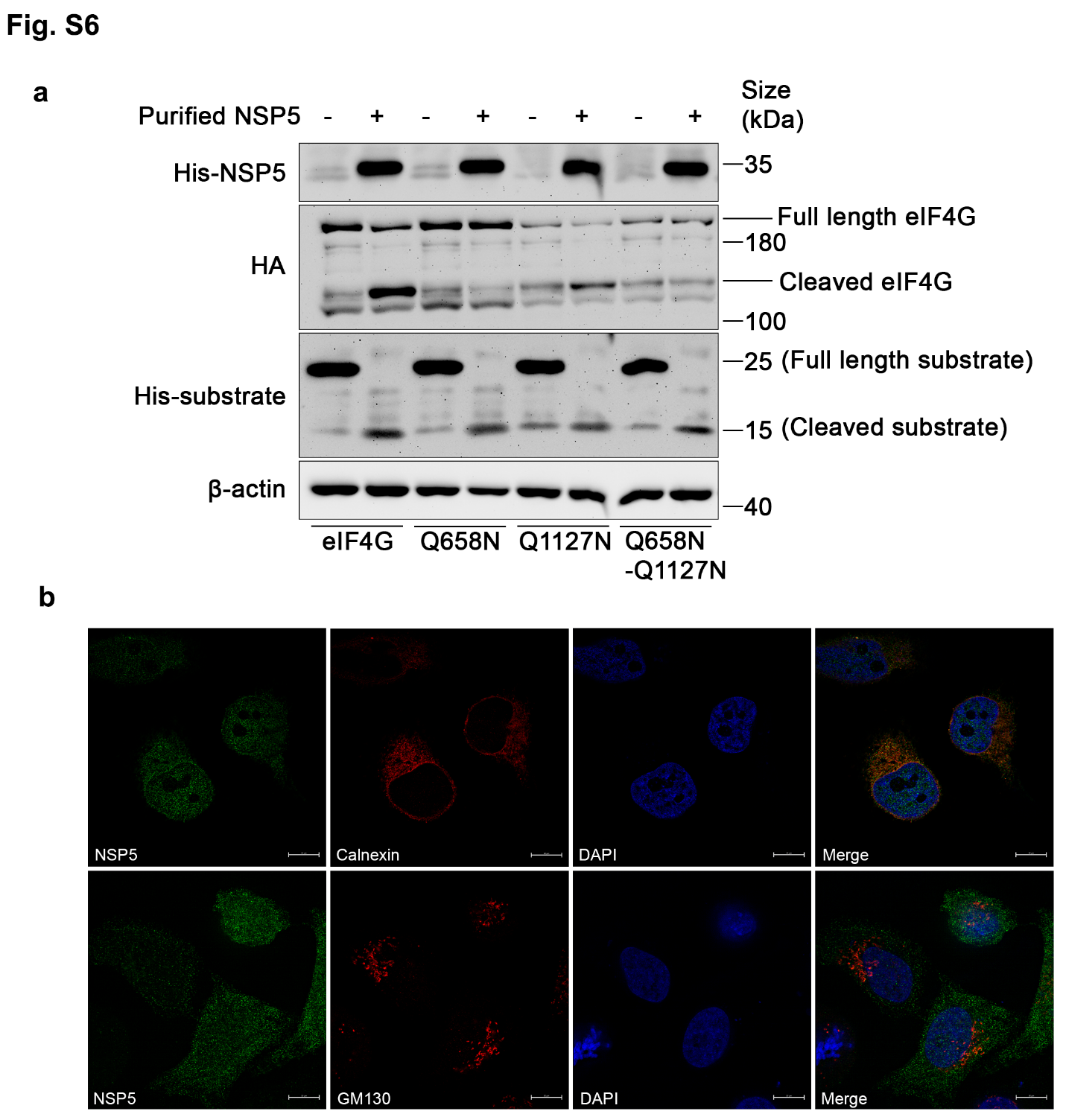
**

**Supplementary Fig. S6 a** WCE from 293T cells overexpressed HA-eIF4G or its mutants was incubated with purified NSP5 (His-tagged). And a His-tag linked control substrate was spiked in as a positive cleavage control. **b** Subcellular locations of NSP5. Immunofluorescence analysis was performed at 24 h after transfection of the plasmid expressing NSP5 into HeLa cells. Calnexin: Endoplasmic Reticulum marker, GM130: Golgi marker. Scale bar = 10 μm.

**Table S1: PCR Primers**

| **Gene** | **Primer** |
| --- | --- |
| β-Actin | Forward ACTGGAACGGTGAAGGTGACA |
|  | Reverse ATGGCAAGGGACTTCCTGTAAC |
| MAVS | Forward AAATTGCCCATCAACTCAACC |
|  | Reverse GGTCTCCTCATTTCTGCTGCT |
| eGFP | Forward GGGCACAAGCTGGAGTACAAC |
|  | Reverse AGTTCACCTTGATGCCGTTCT |
| IFNβ | Forward ATTGCCTCAAGGACAGGATG |
|  | Reverse GCTGCAGCTGCTTAATCTCC |
| IFNλ2/3 | Forward CTGCCACATAGCCCAGTTCA |
|  | Reverse AGAAGCGACTCTTCTAAGGCATCTT |
| ISG15 | Forward GGACCTGACGGTGAAGATGCT |
|  | Reverse ACGCCAATCTTCTGGGTGATCT |
